# High Expression of the Pi-Transporter SLC20A1/Pit1 in Calcific Aortic Valve Disease Promotes Mineralization through Regulation of Akt-1

**DOI:** 10.1371/journal.pone.0053393

**Published:** 2013-01-04

**Authors:** Diala El Husseini, Marie-Chloé Boulanger, Dominique Fournier, Ablajan Mahmut, Yohan Bossé, Philippe Pibarot, Patrick Mathieu

**Affiliations:** 1 Laboratoire d’Études Moléculaires des Valvulopathies (LEMV), Groupe de Recherche en Valvulopathies (GRV), Quebec Heart and Lung Institute/Research Center, Department of Surgery, Laval University, Quebec, Canada; 2 Institut universitaire de cardiologie et de pneumologie de Québec, Quebec, Canada; University of Pecs Medical School, Hungary

## Abstract

The regulation of phosphate (Pi) handling is crucial during calcification of the aortic valve. Gene profiling of Pi transporters revealed that VIC culture expresses SLC201A1/Pit1 and SLC20A2/Pit2. On exposure to a mineralizing medium (2 mM Pi), the expression of Pi transporters in VIC culture is increased several folds, with the highest magnitude for SLC20A1. By using siRNAs, we established that silencing SLC20A1 significantly reduced Pi-induced mineralization of VICs. In human pathological specimens, we found that the expression of SCL20A1 was increased in CAVD tissues compared to control non-mineralized aortic valves. Treatment of VIC culture with Pi promoted the loss of mitochondrial membrane potential (ΔΨm) and cytochrome *c* release within the cytosol, leading to apoptosis. Inhibition of Pi transporters with phosphonoformic acid (PFA) prevented Pi-mediated apoptosis of VICs. Moreover, we discovered that the level of the Akt-1 transcript is diminished in CAVD tissues compared with control valves. Accordingly, treatment with Pi caused a reduction of the Akt-1 transcript in VIC culture, and treatment with PFA or siRNA against SLC20A1 restored the level of Akt-1. Overexpression of Akt-1 (pCMVAkt-1) prevented both Pi-induced apoptosis and mineralization of VIC culture. These results strongly suggest that overexpression of SLC20A1 promotes apoptosis and mineralization by altering the level of Akt-1.

## Introduction

Calcific aortic valve disease (CAVD) is a common disorder of the aging population [1]. Despite the high prevalence of this condition, there is so far no medical treatment for CAVD. To this effect, randomized trials with statins have indicated that a lipid-lowering strategy in patients with CAVD is no more efficient than the placebo [2], [3], [4]. Different risk factors, such as age, male gender, diabetes, and hypertension, have been identified in CAVD [5]. Mineralization of the aortic valve is the major culprit in the development of CAVD. The key molecular processes leading to the mineralization of the aortic valve are just beginning to be understood, and this understanding is of utmost importance in devising novel medical therapies.

Local metabolism and phosphate handling (Pi) are an important pathway in the control of pathological tissue mineralization [6]. Studies indicate that Pi transporters, such as SLC20A1/Pit1 play an important role in the mineralization of blood vessels [7]. To this effect, intracellular channelling of Pi is known to promote mineralization. However, the cellular pathways that are triggered following intracellular entry of Pi remain largely unexplored. It is worth noting that phosphate-generating enzymes are highly expressed during mineralization and elevate the concentration of Pi in the extracellular space. One recent study indicates that the ectonucleotidase enzyme, ectonucleotide pyrophosphatase/phosphodiesterase-1 (ENPP-1), is highly expressed in CAVD and that it contributes to the elevation of extracellular Pi levels in valve interstitial cells (VICs), which are the main cellular component of the aortic valve [8]. It follows that Pi may be channelled into the intracellular space, contributing to the mineralization of the aortic valve. Although it is known that Pi induces mineralization in vascular smooth muscle cells and VICs by promoting apoptosis, the role of Pi transporters in this process, as well as the chain of events leading to programmed cell death, has not yet been fully elucidated [9]. In this study, we hypothesized that Pi transporters play a major role in delivering signals of apoptosis in VICs by altering the level of Akt, a kinase involved in cell survival.

## Methods

### Patients

We examined 50 aortic valves that were explanted from patients at the time of aortic valve replacement for CAVD. Control non-calcified aortic valves (n = 28) with normal echocardiographic analyses were obtained during heart transplant procedures. Patients with a history of rheumatic disease, endocarditis, and inflammatory diseases were excluded. Valves with an aortic valve regurgitation grade >2+ were excluded. Patients with reduced left ventricular ejection fraction (LVEF) (<40%) were excluded. All patients underwent a comprehensive Doppler echocardiographic examination preoperatively. Doppler echocardiographic measurements included the left ventricular stroke volume and transvalvular gradients using the modified Bernoulli equation. The protocol was approved by the local ethical committee and informed consent was obtained from the subjects.

### Immunohistochemical Analyses

Immunohistologic analysis for SLC20A1 was performed using the rabbit antibody anti-SLC20A1 (Novus Biologicals, Oakville, ON, Canada). Slides were then incubated with HRP-conjugated and AEC substrate (Dako, Mississauga, ON, Canada). Tissue sections were counterstained with hematoxylin. Rabbit serum was used as a negative control in immunohistologic experiments.

### Valve Interstitial Cells Isolation and *in vitro* Analyses of Calcification

Human VICs were isolated by collagenase digestion. To promote calcification, cells were incubated for 7 days with a pro-calcifying medium containing: DMEM +5% FBS, 10^−7^ M insulin, 50 µg/ml ascorbic acid and NaH_2_PO_4_ at 2 mM. In some experiments, phosphonoformic acid (PFA) (1 mM) or LY294002 (50 µM) (Sigma, Oakville, ON, Canada) was added as specified. The calcium content was determined by the Arsenazo III method (Synermed, Monterey Park, CA, USA), which relies on the specific reaction of Arsenazo III with calcium to produce a blue complex. The results were measured at 650 nm on the Roche Diagnostics Modular P800 Elecsys (Roche Diagnostics, Laval, QC, Canada). This reaction is specific for calcium. Magnesium is prevented from forming a complex with the reactive. The results were normalized for protein contents and reported as percent changes.

### Real-time PCR

RNA was extracted from valves explanted from 78 patients (50 CAVD and 28 controls). RNA was also extracted from cells during *in vitro* experiments. Total RNA was isolated with RNeasy micro kit from Qiagen (Qiagen, Mississauga, ON, Canada). The RNA extraction protocol was performed according to the manufacturer’s instructions using 100 mg of tissue. The quality of total RNA was monitored by capillary electrophoresis (Experion, Biorad, Mississauga, ON, Canada). One µg of RNA was reverse transcribed using the Quantitec Reverse Transcription Kit from Qiagen. Quantitative real-time PCR (q-PCR) was performed with Quantitec SYBR Green PCR kit from Qiagen on the Rotor-Gene 6000 system (Corbett Robotics Inc, San Francisco, CA, USA). Primers for the following transcripts were obtained from Qiagen (Mississauga, ON, Canada): SLC20A1, SLC20A2, SLC17A1, SLC34A1, SLC34A2, Akt-1, Akt-2, and Akt-3. The expression of hypoxanthine guanine phosphoribosyl transferase (HPRT) was used as a reference gene to normalize the results.

### Transfection of Valve Interstitial Cells with pCMVAkt-1

VICs were seeded in 6-well plates (1×10 ^5^ cells/well) for RNA extraction and in 12-well plates (5×10^4^ cells/well) for analysis of calcification. After 24 hours, the cells were transfected with 1 µg of Akt-1 human cDNA ORF clone incorporated into the vector pCMV6-AC from Origene (Rockville, MD, USA). The transfection was done using the Turbofectin 8 system from Origene. After 48 hours, cells were harvested for RNA extraction or exposed to the mineralizing medium.

### Transfection of Valve Interstitial Cells with siRNAs

VICs were cultured into 12-well plates, at a density of 6×10^4^ cells per well, for determination of calcification, and into 6-well plates, at a density of 1×10^5^ cells per well, for real-time PCR analysis performed in the transfection experiment. VICs were grown in a volume of 1 ml and allowed to adhere overnight in serum-containing antimicrobial-DMEM (5% CO^2^ and 37°C). The next day, VICs were transfected by incubation in a HiPerfect reagent containing 2700 ng siRNA (either negative control or SLC20A1, SLC20A2 and Akt-1 sequences) (Qiagen, Mississauga, ON, Canada). After two days, the medium was changed and a second transfection was done with either negative control or SLC20A1, SLC20A2 and Akt-1 siRNAs. After 24 hours, the transfection medium was replaced by a serum-containing antimicrobial-DMEM calcification medium supplemented with 2 mM NaH_2_PO4 (Sigma, Oakville, ON, Canada). VIC cultures were maintained for seven days in a calcification medium that was changed every two days. A third transfection was done three days after the second transfection, and cells were collected on the seventh day of the mineralization process. The transfection efficiency was verified by reduction of the target gene measured by real-time PCR, using SLC20A1, SLC20A2 and Akt-1 primers (Qiagen, Mississauga, ON, Canada).

### Detection of Apoptosis

Apoptosis was documented in human VIC culture by TUNEL assay using an Apoptag Plus Peroxidase In Situ Apoptosis Detection Kit (Millipore, Billerica, MA, USA). For the quantitative analysis, we counted 300 cells of each well and each condition. Then the percentage of apoptotic cells over the total counted cells was calculated. In some of the experiments with VICs, apoptosis was also confirmed using the Apopercentage apoptosis assay (Biocolor, Carrickfergus, UK), and the apoptosis levels were analysed using Image Pro Plus Version 6.1 image analysis software and expressed as pixel units. In some of the experiments, cyclosporine A (0.05 µM) or PFA (1 mM) were added to the growth medium to inhibit the mitochondrial permeability transition pore (MTP) or Pi transporters respectively.

### Release of Cytochrome *c* in Valve Interstitial Cells

The cells were fixed with paraformaldehyde +0.1% Triton and washed in phosphate-buffered saline (PBS), before being incubated with rabbit antibody against cytochrome *c* (Cell Signaling Technology, Danvers, MA, USA) diluted in 5% BSA/PBS. After being washed, the cells were treated with an autofluorescence eliminator reagent and incubated with a secondary anti-rabbit antibody (Abcam, Cambridge, MA, USA).They were also treated with 4′,6-diamidino-2-phenylindole (DAPI, 1 µg/ml). Images were acquired with an Olympus 1X81-UCB microscope using Image Pro Plus Version 6.1 and processed using ImageJ 1.44p.

### Measurement of the Mitochondrial Membrane Potential (ΔΨm) in Valve Interstitial Cells

Valve interstitial cells were grown in a normal or mineralizing medium for four days, in the presence of 1 mM PFA where indicated. On the fourth day, cells were incubated for 20 minutes with 200 nM of MitoPT TRME (ImmunoChemistry technologies, Bloomington, MN, USA). The medium was replaced with a complete medium without phenol red, to remove excess dye. The cells with intact mitochondrial membrane potential were counted in comparison to the total population. Epifluorescence microscopy was performed with an Olympus IX81 inverted microscope (40X) and an Olympus IEQ camera. Images were corrected for background, and subjected to fast iteration and a fine noise filter using Volocity 6.0.1 (Perkin Elmer).

### Quantification of Akt and pAkt by ELISA

VICs were collected (1 ml from each well, 1.10^6^ per condition) and centrifuged at 12,000 rpm for 10 minutes to pellet off dead cells and debris. The quantification of Akt or pAkt was determined in accordance with the manufacturer’s instructions (EMD Millipore, Billerica MA, USA) and normalized with protein contents.

### Statistical Analyses

Results were expressed as means ±SEM. For continuous data, values were compared between groups with Student t-test or a one-way ANOVA to test the group effect (when more than two groups were compared). Post hoc Tukey analyses were done when the p value of the ANOVA was <0.05. Categorical data were expressed as a percentage and compared with the chi-square test. A p value <0.05 was considered as statistically significant. Statistical analysis was performed with a commercially available software package (JMP IN 8.1).

## Results

### Pi Transporters in Human Valve Interstitial Cells

**Figure 1:**
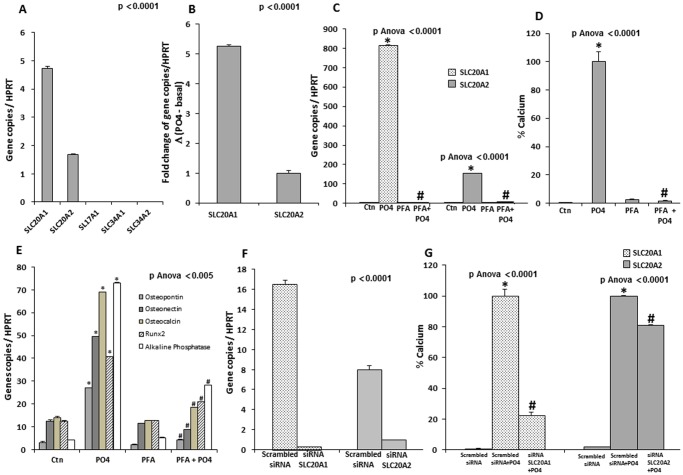
Pi transporters in human valve interstitial cells (VICs). **A**. In isolated VICs, only transcripts of SLC20A1 and SL20A2 were expressed. **B.** Following exposure to Pi, VICs have increased expression of SLC20A1 and SLC20A2 transcripts by several folds, with the highest magnitude for SLC20A1 (results expressed in function of SLC20A2 as the referent) (SLC20A1 increased by 5.3-folds when compared to SLC20A2) **C**. Treatment with PFA, a Pi transporter inhibitor, blocked the rise of SLC20A1 and SLC20A2 transcripts induced by the mineralizing medium (Pi). **D**. PFA prevented the mineralization of VIC cultures induced by Pi. **E**. PFA prevented the Pi-induced rise of osteopontin, osteonectin, osteocalcin, Runx2 and alkaline phosphatase transcripts. **F and G**. In isolated VICs, siRNA-mediated knockdown of SLC20A1 (F) resulted in a decreased Pi-induced mineralization (G). When compared with the knockdown of SLC20A2, the siRNA against SLC20A1 provided a greater reduction of mineralization of VIC cultures (G). (For in vitro experiments n = 3); PFA: Phosphonoformic acid; * p<0.0001 compared to negative control (Ctn); # p<0.005 compared to mineralizing medium (PO_4_).

A gene profiling of potential Pi transporters in human primary VICs, including SLC20A1, SLC20A2, SLC17A1, SLC34A1 and SLC34A2, revealed that only transcripts of SLC20A1 (Pit1) and SLC20A2 (Pit2) were detected (Figure 1A). Of note, the levels of SLC20A1 transcript were 2.5-fold higher than SLC20A2. On exposure to the mineralizing medium (Pi 2 mM), the transcript levels of both SLC20A1 and SLC20A2 increased by several-folds, with the highest magnitude for SLC20A1 (Figure 1B). A treatment with the mineralizing medium did not induce the expression of other phosphate transporters (SLC17A1, SLC34A1, SLC34A2). The addition of PFA, a Pi transporter inhibitor, prevented the rise of SLC20A1 and SLC20A2 mRNA brought about by Pi (Figure 1C). Furthermore, PFA also prevented the mineralization of VIC cultures (Figure 1D) as well as the expression of the bone associated-proteins, osteopontin, osteonectin, osteocalcin, alkaline phosphatase and the master bone transcription factor Runx2 (Figure 1E). VICs were transfected with siRNAs against SLC20A1 and SLC20A2 in order to delineate the role of each Pi transporter on mineralization *in vitro*. In this experiment, both transcripts were reduced by more than 80% by using their respective siRNAs (Figure 1F). While the siRNA for SLC20A2 only slightly reduced mineralization, the siRNA targeting SLC20A1 reduced mineralization of VIC cultures by 78% (Figure 1G). Hence, considering the higher expression of SLC20A1 when compared to SLC20A2 in human VICs, it is likely that the former plays a more important role for Pi transport and mineralization during aortic valve mineralization.

### Expression of SLC20A1 in Human Calcific Aortic Valve Disease

**Figure 2:**
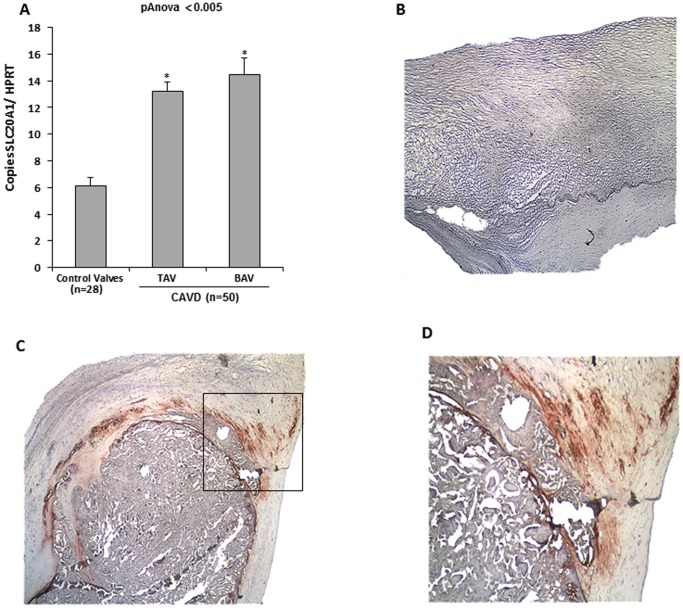
Expression of SCL20A1 in human calcific aortic valve disease. **A**. In human calcific aortic valve disease (CAVD) tissues, expression of SLC20A1 was increased, both in tricuspid and bicuspid aortic valves, when compared to control non-mineralized aortic valves. **B, C and D**. Immunostaining for SLC20A1 revealed faint expression in control aortic valves (B), whereas in CAVD tissues we observed a strong immunostaining in areas of tissue remodelling in the vicinity of calcific nodules (C) (100×) (in panel D magnification 200× of inset in C). * p<0.005 compared to control (Ctn).

Next, the expression level of SLC20A1 in CAVD tissues was examined. Clinical criteria of these patients are presented in Table 1. Compared to control non-calcified aortic valves, the CAVD tissues had significantly more expression of SLC20A1 in both the bicuspid and tricuspid aortic valves (Figure 2A). The expression of the Pi transporter was then confirmed by immunohistochemistry studies, which showed faint expression of SLC20A1 in control non-calcified aortic valves (Figure 2B) and a strong staining in CAVD tissues (Figure 2C and inset in D). In CAVD, immunostaining for SLC20A1 demonstrated that the protein was expressed by cells located at the periphery of mineralized nodules, where the remodelling process is active (Figure 2D).

**Table 1:** Clinical characteristics of patients used for SLC20A1 and Akt-1 quantitative real-time PCR analysis.

	Normal Valve	CAVD	p-value
Age	54.6±9.5	69.9±10.7	0.0001
Male (%)	20	41	NS
Smoking (%)	2	7	0.005
HTA (%)	12	49	NS
Diabetes (%)	6	18	NS
BMI (kg/m^2^)	25.6±5.5	27.8±4.6	0.03
AVA (cm^2^)	–	0.79±0.02	–
Aortic mean gradient(mmHg)	–	43±5	–
BAV (%)	0	18	0.0001
Statins (%)	15	49	NS
TG (mmol/L)	1.56±0.94	1.39±0.63	NS
LDL (mmol/L)	2.30±0.95	2.23±0.96	NS
HDL (mmol/L)	1.21±0.68	1.34±0.35	NS
Creatinine (mmol/L)	113.34±50.70	92.15±25.79	0.02
Creatinine clearance (ml/min)	68.97±26.11	63.60±20.14	NS

CAVD: calcific aortic valve disease; BMI: body mass index; LDL: Low-density lipoprotein; HDL: High-density lipoprotein; TG: Triglycerides; BAV: Bicuspid Aortic Valves.

### Pi Induces Apoptosis of Valve Interstitial Cells through the Mitochondrial Pathway

**Figure 3:**
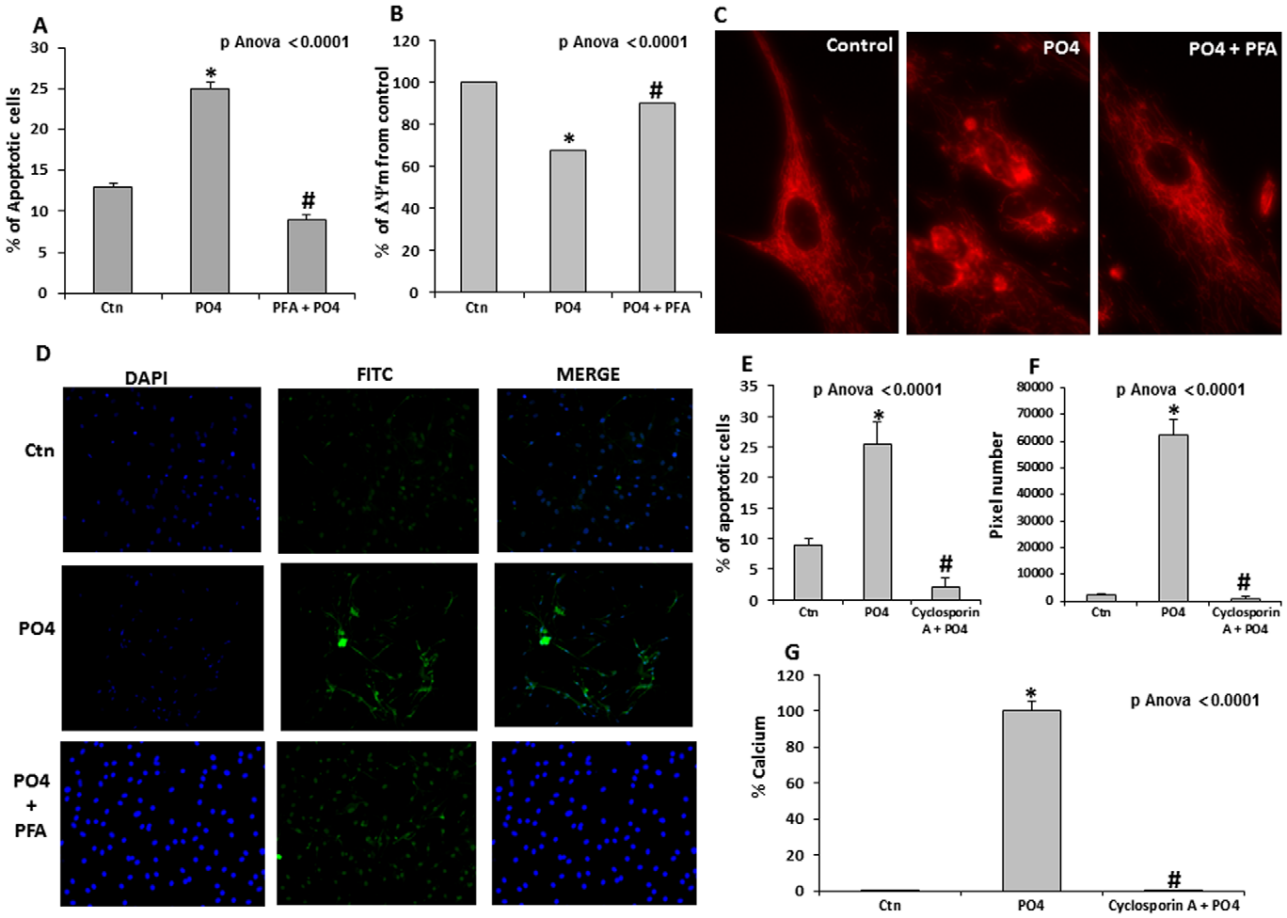
Pi induces apoptosis of valve interstitial cells through the mitochondrial pathway. **A**. The percentage of apoptotic cells, measured by TUNEL assay, increased significantly during VIC mineralization, whereas treatment with PFA blocked this response. **B**. The mitochondrial membrane potential (ΔΨm) decreased following treatment with Pi, but addition of PFA protected the ΔΨm. **C.** Epifluorescence images of VICs with the MitoPT TRME fluorescent dye in different conditions. In control cells, the mitochondrial uptake of MitoPT TRME gives a clear and distinct fluorescent pattern, indicating a normal ΔΨm. Following treatment with Pi, however, the fluorescent pattern is diffuse and accompanied by an abnormal mitochondrial morphology indicating a loss in the ΔΨm. The addition of PFA prevented Pi-mediated loss in the ΔΨm. **D.** This protection with PFA was also confirmed with an immunofluorescence assay measuring cytochrome *c* release in VICs under mineralizing condition. **E**. Cyclosporin A which is an inhibitor of MTP, prevented Pi-mediated apoptosis of VIC cultures as detected with TUNEL assay. **F.** The effect of cyclosporine A on Pi-mediated apoptosis was confirmed by the APOPercentage assay, which relies on changes in membrane asymmetry during apoptosis. **G**. Cyclosporin A prevented Pi-induced mineralization of VIC cultures. (For in vitro experiments n = 3); PFA: Phosphonoformic acid; MTP: mitochondrial permeability transition pore; * p<0.0001 compared to negative control (Ctn); # p<0.0001 compared to mineralizing medium (PO_4_).

To further evaluate the process by which SLC20A1 may promote the mineralization of VIC culture, the level of apoptosis was measured following treatment with a Pi transporter inhibitor, PFA [10]. It should be noted that we had already demonstrated that Pi-induced mineralization of VICs is, to a large extent, dependent on apoptosis [8]. Apoptosis levels were measured by using a TUNEL assay and we demonstrated that PFA completely blocked Pi-induced apoptosis of VICs (Figure 3A). In addition, on exposure to Pi, there was a loss of the mitochondrial membrane potential (ΔΨm) in the VIC culture, which was prevented by PFA (Figure 3B and C). Accordingly, we found that PFA impedes the Pi-mediated cytochrome *c* release within the cytosol of VICs (Figure 3D). Overall, these findings suggest that intracellular channelling of Pi promotes apoptosis of VIC culture by activating the mitochondrial pathway and the opening of the mitochondrial permeability transition pore (MTP). To address the involvement of the MTP, VICs were then treated with cyclosporine A, an MTP inhibitor. Significantly, cyclosporin A inhibited Pi-induced apoptosis of VICs (Figure 3E). These results were also corroborated by the APOPercentage technique, which relies on changes in membrane asymmetry during programmed cell death (Figure 3F). Consequently, cyclosporin A also prevented Pi-mediated mineralization of VICs (Figure 3G).

### Pi-mediated Regulation of mRNA Akt-1 and Mineralization of Valve Interstitial Cells

**Figure 4:**
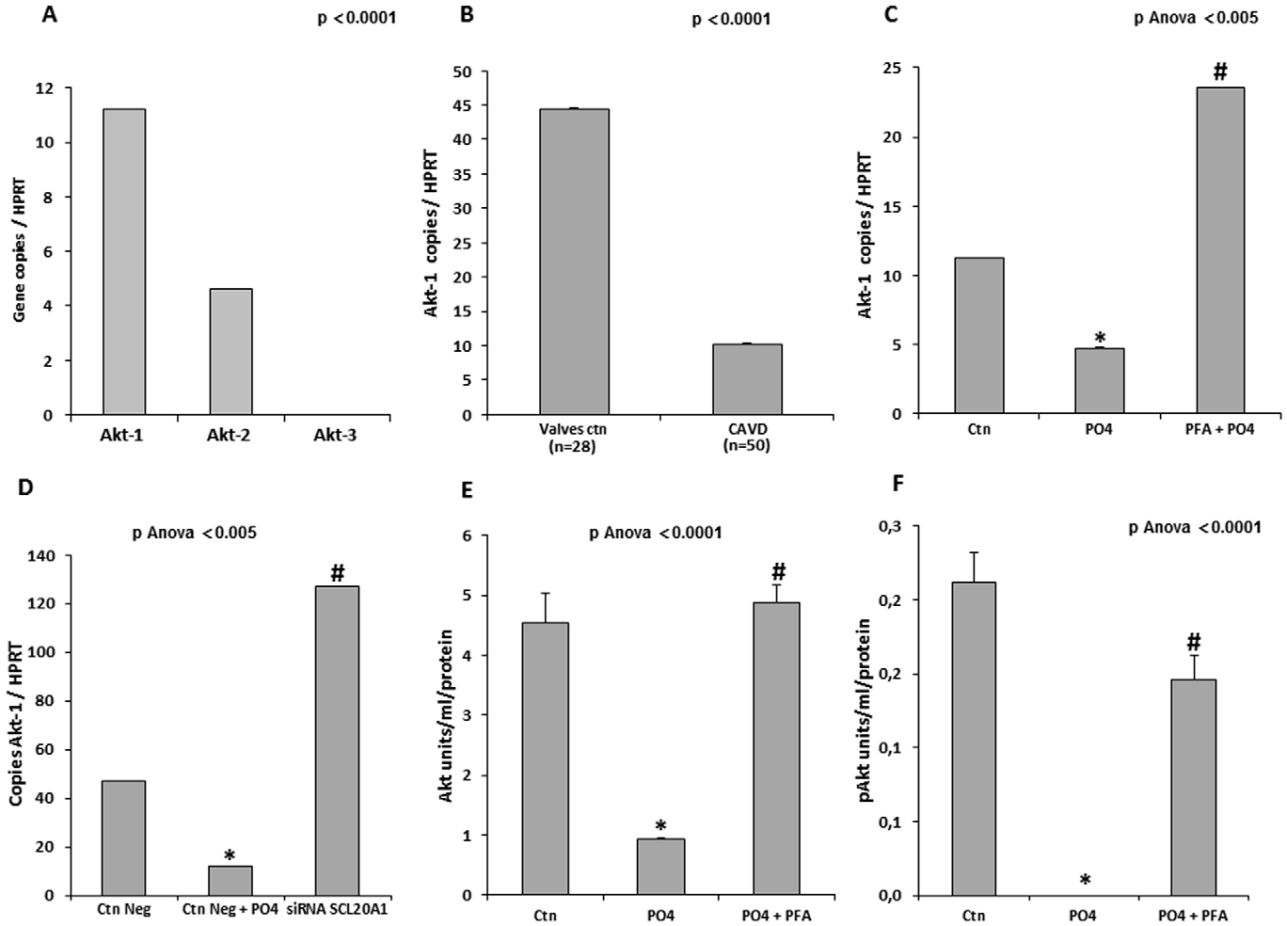
Pi-mediated regulation of Akt levels. **A.** In isolated VICs Akt-1 and Akt-2 were expressed. **B**. The levels of Akt-1 transcript were reduced significantly in CAVD tissues (n = 50) when compared to control non-mineralized aortic valves (n = 28). **C and D.** In isolated VICs, the levels of Akt-1 transcript were lowered following exposure to Pi, whereas PFA (C) and siRNA targeting SLC20A1 (D) prevented this response. **E and F**. Both Akt (E) and pAkt (F) protein levels were reduced by Pi treatment of VICs, whereas in the presence of PFA, levels were maintained. (For in vitro experiments n = 3); PFA: Phosphonoformic acid; CAVD: Calcific Aortic Valve Disease; * p<0.0001 compared to negative control (Ctn); # p<0.005 compared to mineralizing medium (PO_4_).

**Figure 5:**
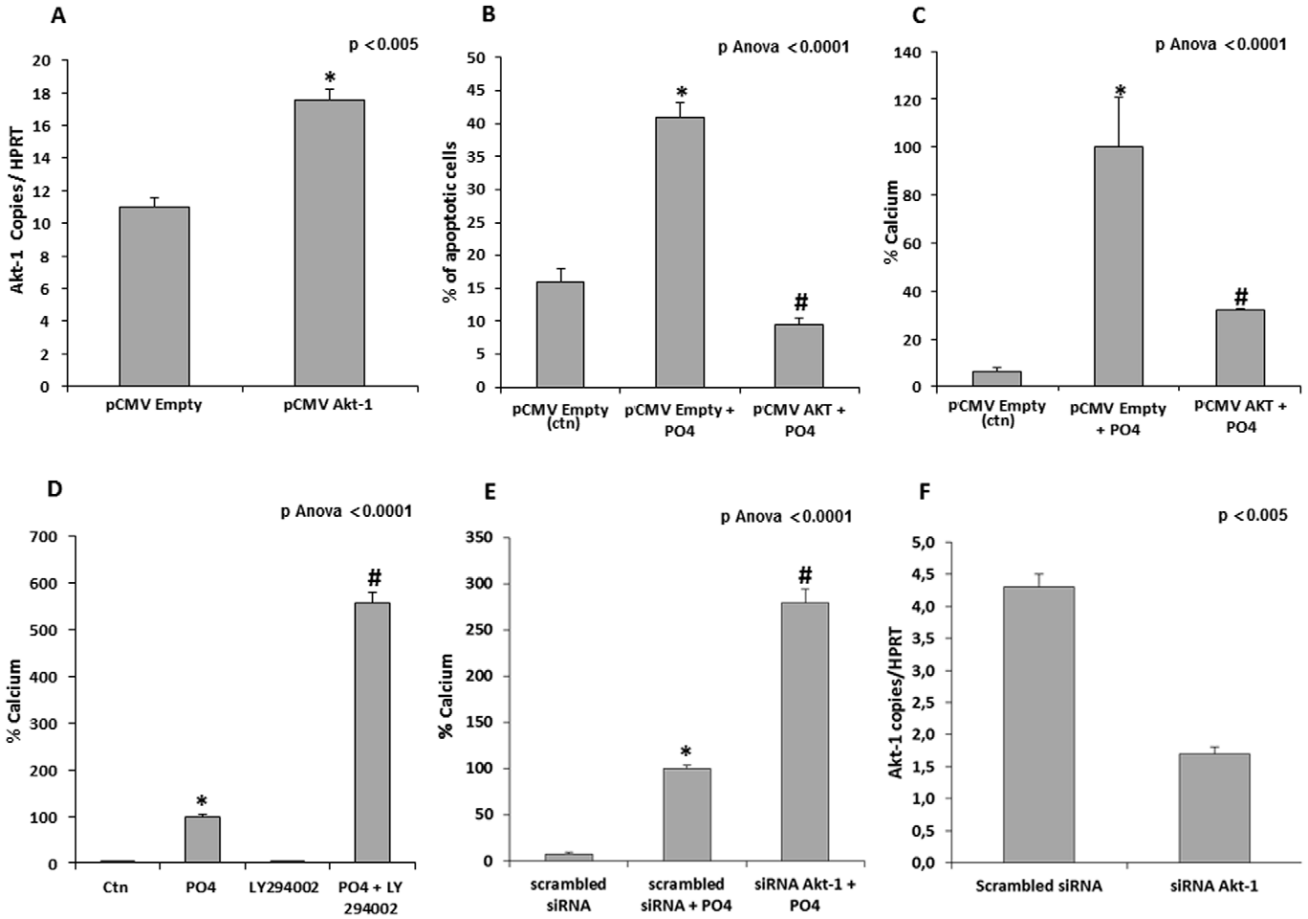
Akt-1 a regulator of Pi-induced mineralization. Transfection of VICs with a pCMVAkt-1 resulted in higher expression of Akt-1 transcripts. **B**. The transfection of Akt-1 (pCMVAkt-1) prevented Pi-induced apoptosis of VICs (measured with the TUNEL assay). **C**. Transfection of Akt-1 reduced significantly Pi-induced mineralization of VIC cultures. **D.** Inhibition of PI3K, a kinase acting upstream of Akt, with Ly294002 increased mineralization of VIC cultures by several-folds. **E and F.** Similarly, the knockdown of Akt-1 (F) resulted in higher mineralization of VIC cultures (E). (For in vitro experiments n = 3); pCMV empty is the control (ctn) in panels A–C * p<0.0001 compared to negative control (Ctn); # p<0.0001 compared to mineralizing medium (PO_4_).

Akt is a kinase involved in cell survival and we thought that it might be involved in apoptosis-mediated mineralization of the aortic valve. Gene profiling of the three genes encoding for Akt demonstrated that mRNAs encoding for Akt-1 and Akt-2 were present in VICs, with Akt-1 being the most highly expressed (Figure 4A). While Akt-2 is involved in glucose metabolism, Akt-1 has been shown to control apoptosis [11]. Measurements of Akt-1 transcript levels in human CAVD tissues indicated that there was a substantial reduction of the transcript levels (4-folds) when compared to control non-mineralized aortic valves (Figure 4B). In VIC culture, we then determined that the Akt-1 transcript levels were lower when the cells were grown in the mineralizing medium (PO4) (Figure 4C). Both PFA and a siRNA targeting SLC20A1 re-established the Akt-1 mRNA levels (Figure 4 C and D). On that account, measurement of the Akt protein levels by ELISA indicated that on exposure to the mineralizing medium, the levels of Akt and phosphorylated Akt (pAkt) were reduced (Figure 4E and F). Treatment of VIC culture with PFA prevented the decrease of Akt and pAkt levels (Figure 4E and F). In addition, VICs were transfected with pCMVAkt-1 (Figure 5A) and apoptosis and mineralization were measured. Overexpression of Akt-1 prevented Pi-mediated apoptosis and mineralization (Figure 5B and C). Conversely, inhibition of PI3K, a kinase acting upstream of Akt, with LY294002 led to a significant rise in the mineralization of the VIC cultures (Figure 5D). Similarly, siRNA against Akt-1 (Figure 5F) increased the mineralization of VIC cultures by 2.6-folds (Figure 5E). When taken together, these results indicate that the cellular entry of Pi can modulate the expression of Akt-1, which constitutes an important target in apoptosis-mediated mineralization of the aortic valve.

## Discussion

This is the first study to demonstrate that SLC20A1 is highly expressed in CAVD tissues and that it promotes mineralization of VICs by altering the level of mRNA encoding for Akt-1. Specifically, we found that Pi promotes apoptosis of VICs by altering the ΔΨm, the formation of the MTP and the release of cytochrome *c* within the cytosol. In addition, we observed that Pi-mediated down-regulation of Akt-1 is a key event in the process leading to programmed cell death and the mineralization of VICs. These findings may therefore have a significant impact on our understanding on how local handling of Pi by VICs promotes mineralization of the aortic valve.

### SLC20A1/Pit1 is Highly Expressed in Calcific Aortic Valve Disease: Functional Relevance

The present findings highlight that SLC20A1 is highly expressed in CAVD. The localization of SLC20A1 in the vicinity of calcified nodules suggests that the Pi transporter might play a role in the control of mineralization in CAVD tissue. Moreover, by using gene-profiling studies, we established that among the different Pi transporters, SLC20A1 is the most highly expressed. In addition, on exposure to Pi, the expression of SLC20A1 transcripts was increased by several-folds, indicating that there is a positive feedback loop between the availability of Pi and the transporter expression. It is worth noting that SLC20A2 was also expressed but to a lower level compared to SLC20A1. SLC20A1 and SLC20A2 knockdown demonstrates that the reduction of SLC20A1 induces a significant decrease in VIC mineralization. This finding is consistent with the notion that the level of Pi transporters expressed in a tissue is the main limiting factor that will modulate the channelling of Pi within the intracellular space [12].

### Pi-mediated Apoptosis Relies on the Mitochondrial Pathway

The expression of SLC201A is ubiquitous and its role in the mineralization of the vascular wall and smooth muscle cells has been previously highlighted [13]. Nevertheless, the precise mechanism by which cellular entry of Pi mediates the mineralization of VICs has yet to be clearly delineated. Previous investigations with vascular smooth muscle cells have revealed that Pi-mediated mineralization is accompanied by the expression of bone-related proteins [14]. In this regard, the present study also emphasized that on exposure to Pi, VICs expressed several bone-related proteins, including Runx2, a master bone transcription factor. Furthermore, we documented that inhibiting SLC20A1 with PFA prevented the rise of bone-related transcripts induced by the mineralizing medium. It is worth noting that despite the expression of bone-related proteins, Pi-mediated mineralization of both vascular smooth muscle cells and VICs relies mainly on apoptosis [15]. In this regard, the level of apoptosis in human pathological CAVD specimens is high, and the inhibition of caspases prevents Pi-induced mineralization of VIC cultures [8]. This raises an important question: By which mechanism does Pi transport within the cell contribute to apoptosis-mediated mineralization of the aortic valve? To answer this, we observed, in VIC culture, that Pi-induced apoptosis was dependent on the mitochondrial pathway. Specifically, Pi promotes a loss of the ΔΨm which is associated with cytochrome *c* release. The release of cytochrome *c* from mitochondria within the cytosol is a key process in the activation of effector caspases during programmed cell death [16]. It should be pointed out that the inhibition of Pi transporter with PFA prevented ΔΨm loss, cytochrome *c* release and consequently Pi-mediated apoptosis of VIC cultures. These findings suggest that MTP opening might be involved in the mineralization of VIC cultures. Accordingly, we found that inhibiting the MTP with cyclosporine A prevented Pi-mediated apoptosis and the mineralization of VIC cultures. Overall, these findings suggest that Pi-induced mineralization of VIC culture is dependent on the intracellular channelling of Pi, whereby apoptosis is triggered through the mitochondrial pathway.

### Pi-mediated Mineralization of Valve Interstitial Cells is Dependent on Akt-1

VIC survival is largely dependent on the PI3K/Akt pathway. In the present study, we established that mRNA levels of Akt-1 were significantly reduced in VIC culture following treatment with Pi. Moreover, Akt-1 transcript levels were severely down-regulated in CAVD tissues when compared to control non-mineralized aortic valves. In the same line, a recent study documented that Akt signalling is down-regulated in mineralized aortic valves [17]. Interestingly, *in vitro*, both PFA and a siRNA against SLC20A1 prevented Pi-mediated down-regulation of Akt-1 mRNA transcript levels, indicating that this transporter is involved in the regulation of the PI3K/Akt pathway. Reduced level of Akt-1 at the mRNA level was associated with a decrease in the protein level of phosphorylated Akt (pAkt). Overall, these findings suggest that the regulation of VIC apoptosis and mineralization is dependent on the level of Akt-1. This latter hypothesis was reinforced by the discovery that the overexpression of Akt-1 in VIC cultures prevented Pi-mediated apoptosis and mineralization. Although the mechanism by which Pi modulates Akt-1 mRNA levels is not yet fully elucidated, it is possible that Pi affects transcription and/or mRNA stability.

### Limitations

This study examined CAVD tissues with advanced pathological mineralization, but we cannot necessarily transpose these findings to early processes involved in the development of aortic sclerosis. Nevertheless, the present findings highlight the role of Pi transporters and Akt-1 in the process of aortic valve mineralization. In this study, we used PFA as a pharmacological inhibitor of Pi transport. Although PFA was criticized as being a potentially weak inhibitor of SLC20A1 in one study [18], other reports using a longer incubation time of the inhibitor in cultured cells have documented that PFA in fact significantly reduces the intracellular channelling of Pi [19]. Specifically, Jono et al. showed that the incubation of human vascular smooth muscle cells with PFA (1 mM) for several days reduced Pi uptake by 75%. Under similar conditions, we found that the Pi-mediated expression of osteoblast genes [20] and the mineralization of VIC cultures were inhibited by the addition of PFA for 7 days. Nonetheless, in the present study, we established some important findings by decreasing the SLC20A1 level. In this regard, we have conclusively shown that Pi-induced mineralization and the down-regulation of Akt-1 were abrogated by both PFA and the knockdown of SLC20A1. Hence, these findings indicate that both the expression level and functional properties of SLC20A1 are important in regulating the mineralization of the aortic valve.

### Conclusion

This study provides evidence that SLC20A1 is overexpressed in CAVD tissue and that it contributes to mineralization by modifying the level of Akt-1. Pathological mineralization of aortic valve may be, at least in part, dependent on the intracellular channelling of Pi, whereby the mitochondrial-dependent apoptosis pathway is triggered. Further research on Pi transport in CAVD may help to develop novel therapies based on Pi handling.
